# Developing customized stepwise MIRU-VNTR typing for tuberculosis surveillance in Georgia

**DOI:** 10.1371/journal.pone.0264472

**Published:** 2022-03-01

**Authors:** Nino Maghradze, Levan Jugheli, Sonia Borrell, Nestani Tukvadze, Russell R. Kempker, Henry M. Blumberg, Sebastien Gagneux

**Affiliations:** 1 Swiss Tropical and Public Health Institute, Basel, Switzerland; 2 University of Basel, Basel, Switzerland; 3 National Center for Tuberculosis and Lung Diseases (NCTLD), Tbilisi, Georgia; 4 Division of Infectious Diseases, Department of Medicine, Emory University School of Medicine, Atlanta, GA, United States of America; 5 Departments of Epidemiology and Global Health, Rollins School of Public Health of Emory University, Atlanta, GA, United States of America; North Eastern Regional Institute of Science and Technology, INDIA

## Abstract

**Introduction:**

Mycobacterial Interspersed Repetitive Units–Variable Tandem Repeats (MIRU-VNTR) typing has been widely used for molecular epidemiological studies of tuberculosis (TB). However, genotyping tools for *Mycobacterium tuberculosis* (Mtb*)* may be limiting in some settings due to high cost and workload. In this study developed a customized stepwise MIRU-VNTR typing that prioritizes high discriminatory loci and validated this method using penitentiary system cohort in the country of Georgia.

**Methods:**

We used a previously generated MIRU-VNTR dataset from recurrent TB cases (32 cases) in Georgia and a new dataset of TB cases from the penitentiary system (102 cases) recruited from 2014 to 2015. A Hunter-Gaston Discriminatory Index (HGDI) was calculated utilizing a 24 standard loci panel, to select high discriminatory power loci, subsequently defined as the customized Georgia-specific set of loci for initial typing. The remaining loci were scored and hierarchically grouped for second and third step typing of the cohort. We then compared the processing time and costs of the customized stepwise method to the standard 24-loci method.

**Results:**

For the customized Georgia-specific set that was used for initial typing, 10 loci were selected with a minimum value of 0.32 to the highest HGDI score locus. Customized 10 loci (step 1) typing of 102 Mtb patient isolates revealed 35.7% clustered cases. This proportion was reduced to 19.5% after hierarchical application of 2^nd^ and 3^rd^ step typing with the corresponding groups of loci. Our customized stepwise MIRU-VNTR genotyping approach reduced the quantity of samples to be typed and therefore overall processing time and costs by 42.6% each.

**Conclusion:**

Our study shows that our customized stepwise MIRU-VNTR typing approach is a valid alternative of standard MIRI-VNTR typing panels for molecular epidemiological investigation in Georgia that saves time, workload and costs. Similar approaches could be developed for other settings.

## Introduction

Genotyping plays an important role in the surveillance and molecular epidemiology of tuberculosis (TB). However, the implementation of TB genotyping methods has been limited in many settings, especially in low- and middle-income countries (LMIC), given that they can be computationally demanding, time-consuming and costly [1, 2]. Mycobacterial Interspersed Repetitive Unit-Variable Number of Tandem Repeats (MIRU-VNTR) typing is widely used to evaluate the population structure, strain genetic diversity, and transmission of the *Mycobacterium tuberculosis* complex (MTBC) [3, 4]. Globally, the human-adapted MTBC can be classified into nine phylogenetic lineages that differ in their geographic distribution [5, 6]. This phylo-geographic genetic diversity also impacts the discriminatory power of MIRU-VNTR loci in a given population, which has led to the development of customized MIRU-VNTR loci sets for application in different geographical settings [2, 7, 8]. Compared to the MIRU-VNTR typing using standard 12, 15 or 24 loci panels proposed by Supply *et al* [9], customized approaches have focused on the most sensitive and highly discriminatory loci [10, 11], allowing for reduced workload, labor and consumable-dependent cost and sample processing time.

However, customization and reduction of the MIRU-VNTR loci set, might be inefficient for reaching higher level of discrimination, especially where the variety of MTBC lineages is limited and the bacterial population is homogeneous. In such instances, various combinations of genotyping tools for increasing resolution have been proposed [11–13], but no systematic approach has yet been established. Considering the limited resources in LMIC, combining several distinct typing methods increases the complexity and costs, and the absence of standardization results in poor comparability between the laboratories. In the present study, we implemented customized stepwise MIRU-VNTR typing specifically for the epidemiological setting of Georgia [14]. This approach is based on selecting and developing groups of MIRU-VNTR loci, to reduce the number of samples to be typed after each step.

Recently, standard 24-loci MIRU-VNTR typing has been established at the National Reference Laboratory (NRL) of National Center of Tuberculosis and Lung Diseases (NCTLD) in Tbilisi, Georgia, which has been used to differentiate between relapse and reinfection in recurrent TB cases [15]. In this study, we modified the standard 24-loci MIRU-VNTR panel into a customized stepwise typing approach for the Georgian Mtb population and applied it to a patient population from the penitentiary system.

While important progress in TB control and a substantial decrease in TB incidence in Georgia from 2002 (228 per 100,000) to 2019 (80 per 100,000) has occurred, many challenges to TB elimination remain, including high rates of drug-resistant TB cases. In particular, the penitentiary system has been identified as a hotspot of TB disease transmission including multidrug-resistant (MDR)-TB [16–18]. Over the past six years, with the advent of new programmatic initiatives, cases of TB diagnosed in the penitentiary system have decreased, including MDR and extensively drug-resistant (XDR) TB, from 33% of all MDR cases in the country in 2011 to the 4.1% in 2020 (National Surveillance Program, unpublished data). However, the penitentiary system remains a persistent site for transmission of drug-resistant TB [17–19], bearing a risk of infection spillover into the community. Hence, rapid detection of TB transmission in prisons, along with infection surveillance in the country, is crucial, and resource-adapted molecular methods for surveillance are therefore needed.

## Materials and methods

### Ethics

Ethical approval for the study was obtained from the relevant Institutional Review Boards (IRB) of the NCTLD in Georgia and in Switzerland (Ethikkommission Nordwest- und Zentralschweiz). Due to observational nature of the study which do not involve any physical intervention and was solely using the routinely collected samples from TB patient in Georgia, the need of informed consent was waived by the National Council on Bioethics of Ministry of Labor, Health and Social Affairs of Georgia, and therefore by local IRB. The data from archived samples were fully anonymized to guarantee the confidentiality.

### Customization and validation of country-specific minimal set of MIRU-VNTR loci

#### Genotyping dataset and parameters for customization

In order to develop a customized loci set for the Mtb strain population in Georgia, we first re-analyzed a dataset from a previous study of recurrent TB cases, with a retrospective cohort of 32 patients (64 samples) from 2014–2016, typed with the standard 24 loci panel [15]. Inclusion criteria of the samples for the relapsed cases was availability of the samples in the National Reference Laboratory biobank. From this dataset, we used allelic data for 37 samples; 27 single Mtb isolates from relapsed patients and paired isolates from 5 reinfection cases. We defined genotypic clusters with a single locus variants (SLV), and calculated discriminatory indexes for each locus of the 24-loci panel using Hunter and Gaston discriminatory index (HGDI) [20]. Based on the Simpson’s index of diversity, HGD index was calculated using the online tool http://insilico.ehu.es/mini_tools/discriminatory_power. Following the HGDI calculations and acknowledged cut-offs, the loci were classified into high (HGDI≥0.6), moderate (0.3≤ HGDI<0.6) and low (HGDI<0.3) discriminant categories [18]. We ranked the 24 loci based on their HGDI score and selected the 10 loci with highest discriminatory indexes.

#### Genotyping dataset for customized set validation

For the purpose of validating our customized set of loci, we focused on patients with pulmonary TB residing and diagnosed in the penitentiary system in 2014–2015. Cases were not overlapping the recurrent TB case samples and were identified through the national TB surveillance database and were linked to the National Reference Laboratory (NRL) sample ID numbers to identify the NRL strain biobank and phenotypic drug susceptibility testing (pDST) results. We were able to link 136 TB cases in prisons from 2014.2015, with bio banked Mtb isolates for further use in the study, however, total of 102 Mtb isolates were recovered from the laboratory biobank and were typed using the customized 10 loci set. Specifically, the 10 customized MIRU-VNTR loci were multiplexed in four separate PCRs– 1) MIRU16, MIRU31 and ETRB; 2) MIRU40, MIRU26 and Mtub30; 3) Mtub 21, QUB11b and QUB26; 4) MIRU39. Negative (H_2_O) and positive (H37Rv DNA) controls were included in each reaction. PCR amplification was performed under following conditions: the initial denaturation at 95°C for 15 minutes, followed by 40 cycles of 94°C for 1 minute, 59°C for 1 minute and 72°C for 1.5 minutes with final extension at 72°C for 10 minutes. We used low-resolution electrophoresis for amplification check, followed by higher resolution electrophoresis with 1.8% agarose gel, using 1kb ladder as size marker. A numerical code was obtained by comparison of the allelic table published by Supply *et al* [16]. Genotypic clusters were defined based on SLV.

### Re-evaluation of the customized set and HGD indexes and stepwise approach development

**Fig 1:**
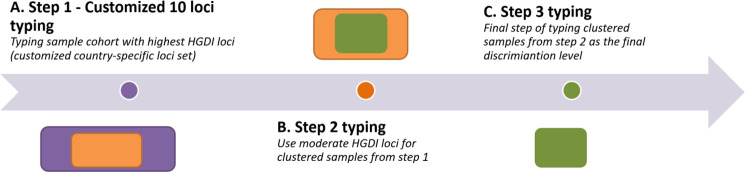
Schematic representation of the MIRU-VNTR stepwise approach. Schematic representation of the stepwise approach, with reduced amount of the samples to be typed after application of each step of locus group, resulting reduced time, cost and workload.

For the stepwise typing approach development, we used the remaining 14 loci to type 35 Mtb isolates from the penitentiary system that were clustered after typing with the customized 10 loci set. Additionally, we re-assessed the discriminatory power of each locus based on the combined datasets of i) recurrent TB cases (37 samples) and ii) prison cohort samples for which full 24 loci results were available (35 samples). The HDGI was re-calculated and the loci were ranked following the cut-off scores described above. The criteria of locus hierarchy and inclusion into the two-step typing panel was defined by i) HGDIs, calculated within the recurrent TB cases and penitentiary system cohort; and ii) ability of the locus to discriminate and reduce proportion of the samples to be typed by the stepwise addition of each locus to the predefined 10 loci customized set (Fig 1).

### Cost and time assessment

Along with method modification, we assessed the processing time and approximate direct cost based on the technician per-hour labor cost, consumables and reagents pricing in Georgia for a batch of 54 samples processed with the customized stepwise MIRU- VNTR typing (excluding DNA extraction) and standard 24-locus panel, with 2 additional controls. The number of samples in a batch was defined based on the number of wells available on our electrophoresis tray.

## Results

### Customization of the Georgia-specific MIRU-VNTR loci set using typing results from recurrent TB cases

**Fig 2:**
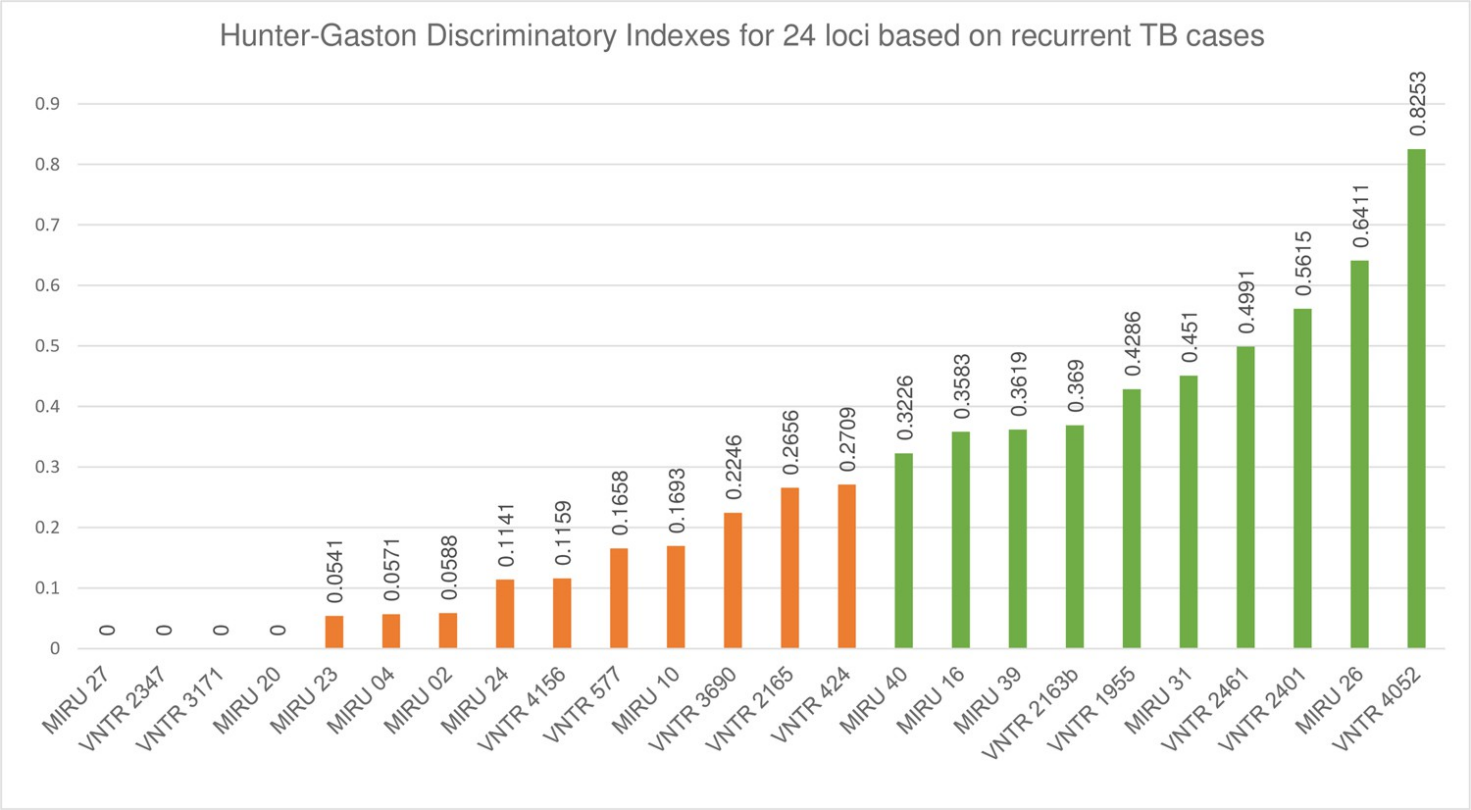
HGDI scores of 24-loci panel based on recurrent TB cases. Graph represents individual discriminatory index for 24-loci panel generated from recurrent TB cohort typing results. Green bars indicate “the Georgian customized set” with high and moderate index loci included in the customized set.

Utilizing the standard 24-loci panel, MIRU-VNTR typing of 37 samples from our previously published cohort of recurrent TB cases [15] revealed two highly discriminatory loci, namely—VNTR4052 (QUB-26) and MIRU26, with an HGDI of 0.83 and 0.64, respectively (Fig 2). Eight moderately discriminant loci were identified: VNTR2401/Mtub30 (HDGI = 0.56), VNTR2461/ETR B (HGDI = 0.50), MIRU31/ETR E (HGDI = 0.45), VNTR1955/Mtub21 (HGDI = 0.43), VNTR2163b/QUB-11b (HGDI = 0.34), MIRU39 (HGDI = 0.36), MIRU16 (HGDI = 0.36), MIRU40 (HGDI = 0.32). Another ten loci showed low discriminatory indices: VNTR424/Mtub04 (HGDI = 0.27), VNTR2165/ETR A (HGDI = 0.27), VNTR3690/Mtub39 (HGDI = 0.22), MIRU10 (HGDI = 0.17), VNTR577/ETR C (HGDI = 0.17), VNTR4156/QUB-4156c (HGDI = 0.12), MIRU24 (HGDI = 0.11), MIRU02 (HGDI = 0.06), MIRU04/ETR D (HGDI = 0.06) and MIRU23 (0.05). Finally, four loci, MIRU20, VNTR2347/Mtub 29, MIRU27/QUB-5 and VNTR3171/Mtub34 showed no discriminatory power with an HGDI score of zero (Fig 2). A Georgia-specific panel of ten loci was defined, which included the two highly and the eight moderately discriminatory loci (Fig 2). The loci in the customized Georgia-specific set were compared to the standard 15- and 12-loci panels [2]. Our customized panel shared four loci with both the 12- and 15-loci standard panels, while four and one loci were exclusively shared with the 15-loci set and the 12-loci set, respectively. VNTR2461 with a moderate discriminatory power in the Georgia-specific panel has so far not been included in earlier proposed typing sets.

### MIRU-VNTR typing of the Mtb samples from the penitentiary system

#### Genotyping with the customized 10-loci set

**Fig 3:**
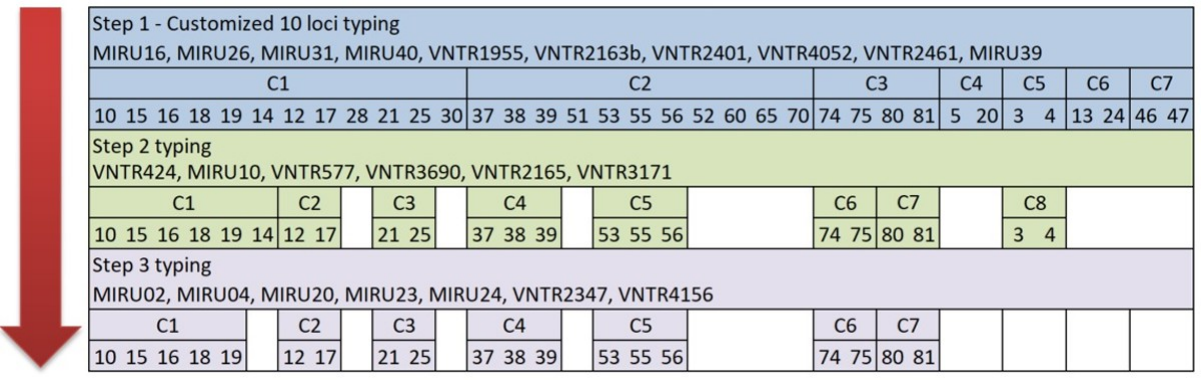
Stepwise application of customized and additional 14 loci and distribution of the clustered samples. Selected loci for the stepwise approach and distribution of the clustered samples after typing with customized 10-loci set, step two typing with 6 loci and step 3 typing with remaining 8 loci.

From total of unique 102 Mtb isolates from prison TB cases, full allelic data was obtained for 98 (96.1%) samples. MIRU-VNTR genotyping of 98 Mtb isolates with our customized 10-loci set revealed seven clusters by SLV comprising 35 clustered isolates and 63 singletons (non-clustered isolates). The 35 clustered isolates were distributed in the seven clusters as follows: cluster 1–12 isolates (34.4%), cluster 2–11 (31.4%), cluster 4–4 (11.4%), cluster 4 to 7 were represented with two (5.7%) isolates per cluster (Fig 3).

For the stepwise typing approach development of the clustered isolates, we used the remaining 14 loci from the standard 24-loci panel not included in our customized 10-loci set. These resulted in an additional 16 singletons, while the number of clusters remained the same, with a sample distribution as follows: cluster 1 –five isolates (26.3%), cluster 2, 3, 6 and 7 each with two isolates (10.5%), while cluster 4 and 5 had three (15.8%) isolates each, resulting in a total of 19/98 (19.5%) clustered isolates (Fig 3).

#### Defining loci sets for the stepwise approach

**Fig 4:**
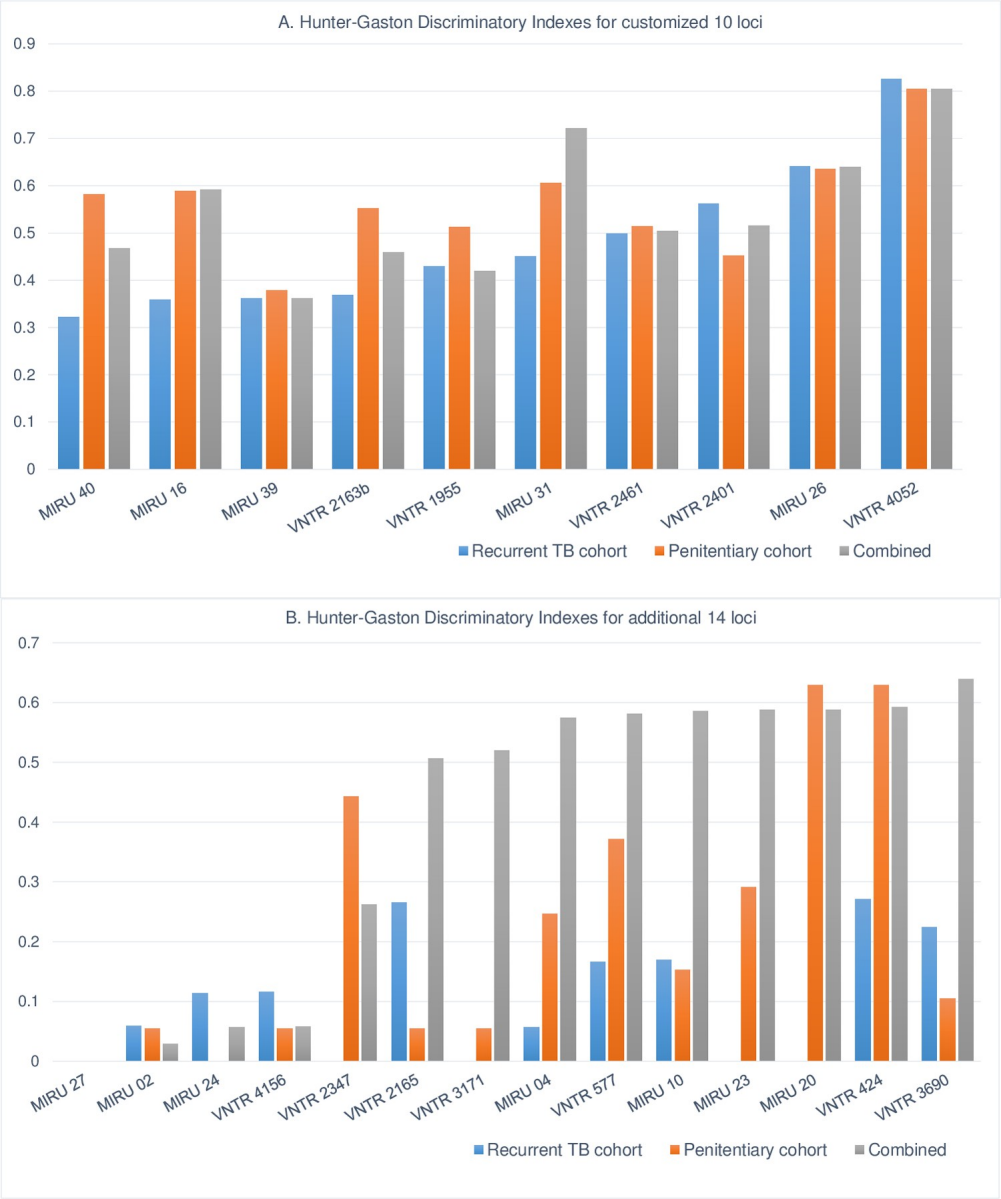
Individual locus HGDI of 10 customized and additional 14 loci. (A) HGDI calculated for the customized 10 loci and (B) the additional 14 loci, based on typing of recurrent TB cases (civil cohort) in blue bars along with same indexes calculated within penitentiary system samples in orange. Grey bars indicate indexes calculated from combined datasets with 24 loci typing. Category definition: HGDI≥0.6 high discriminatory power, 0.3≤HGDI≤0.6 moderate discriminatory power, HGDI<0.3 low discriminatory power.

In order to combine loci for the second typing step, we re-evaluated and compared rankings of HGDI scores (Fig 4A, 4B) by assessing the number of discriminated samples by each locus added separately to the customized 10-loci set within the combined dataset. VNTR424/Mtub04 showed an increased HGDI (within the additional 14 loci set) in the penitentiary system compared to recurrent TB typing data (Fig 4B). Four loci–MIRU10, VNTR577/ETR C, VNTR2165/ETR A and VNTR3690/Mtub39 added most to the increase in HGDI. VNTR3171/Mtub34 showed an increased capacity to discriminate between the samples when added to these four loci, while the HGDI of VNTR3171/Mtub34 on its own was 0.05 and 0 in the penitentiary and recurrent TB cohort was, respectively. Application of the 2^nd^ step loci VNTR424/Mtub04, MIRU10, VNTR577/ETR C, VNTR2165/ETR A, VNTR3690/Mtub39, and VNTR3171/Mtub34 reduced the number of clustered isolates from 35 to 22, leading to the reduction of the samples to be genotyped by 37%.

The remaining eight loci (MIRU02, MIRU04/ETR D, MIRU20, MIRU23, MIRU24, MIRU27/QUB-5, VNTR2347/Mtub 29, and VNTR4156/QUB-4156c) were included in a third and last step of genotyping. The HGDI of MIRU27/QUB-5 remained zero in both cohorts as it did not identify any discrete sample from the population. Application of the third step loci on 22 clustered samples identified 2 singletons, defining the final outcome of the customized stepwise MIRU-VNTR typing of penitentiary system cohort– 19 (19.4%) clustered cases from the total of 98 samples.

### Processing time for the customized stepwise MIRU-VNTR typing

Considering a conventional method with two gel trays with 32-well gel comb, it is possible to run 54 samples with 2 controls and 8 molecular size markers. To compare the total amount of time required for our customized stepwise genotyping approach and for the standard 24-loci method, we calculated the processing time for 56 samples (including controls) for both methods. Our calculation included the following steps: master mix preparation (20 min.), adding sample DNA (20 min.), PCR (165 min.), and loading samples for gel electrophoresis (40 min.), running the gel (240 min), dying (20 min.) and imaging (8 min).

Applying the full 24-loci panel on the 102 patient isolates resulted in a total of 2,448 amplicons to run on gel electrophoresis, with a total processing time of 387.6 person-hours. By contrast, our customized stepwise typing approach resulted in a reduced number of amplicons to be processed from 2,448 to 1,020 (41.7%) after 1^st^ step customized 10 loci typing. This was followed by 210 (8.6%) amplicons after applying the 2^nd^ step typing with 6 loci, and to 176 (7.2%) amplicons to be typed for the 3^rd^ step with the remaining 8 loci. Hence, our customized stepwise approach resulted in a total of 1,406 amplicons to run, with 41.7% and 8.6% reduction of typing with 1^st^ step customized loci and 2^nd^ step typing with an additional 6 loci, respectively. Compared to the 387.6 person-hours with 24-loci panel typing, the stepwise approach required 222.6 person-hours, which corresponds to a reduction in total processing time of 42.6%.

### Cost comparison of the customized stepwise genotyping approach

The price of the 24-loci MIRU-VNTR per sample based on consumable prices in Georgia in addition to laboratory technician salary per-hour was approximately 1,124 Euros, while the customized stepwise approach using the 10-6-8 loci panels sequentially resulted in a decreased cost of approximately 648 Euros. This corresponds to a cost reduction of 42.6% compared to the standard method, indicating a major dependence of the total cost on the sample quantity and processing time.

## Discussion

We adapted the standard 24-loci MIRU-VNTR panel for the Georgian TB population by i) reducing the number of loci to a customized set of 10 loci for an initial step of genotyping; ii) developing a stepwise typing approach to retain adequate discriminatory power; iii) applying this approach on a retrospective TB patient cohort in a penitentiary system in Georgia. Our findings demonstrate that the customized stepwise MIRU-VNTR tying approach reduces the processing time, cost and workload, while achieving equal discriminatory power to 24 standard MIRU-VNTR loci panel. The modified MIRU-VNTR typing can be a benefit for the TB disease transmission control in the most effective way, especially for LMIC.

For the purpose of the MIRU-VNTR customization, we modified the 24-loci panel based on the discriminatory power of each loci using HGDI. Initially, we chose the 10 highest HGDI score loci and compared them to the standardized 12- and 15-loci panel sets (Table 1). While four loci included in our customized set were shared with both the standard sets, one locus was shared only with the 12-loci panel, four loci only with the 15-loci panel, and one locus—VNTR2461 (ETRB) was not present either of the two standard sets. The bias of 15-loci and 24-loci panels towards MTBC lineage 4 has been well-documented [9]. In Georgia, most MTBC belongs to lineage 4 and lineage 2/Beijing. Deviation from the standard loci panels is mostly influenced by HGDI sensitivity to local strain variation. Therefore, our results from assessing a customized set, based on the MTBC lineages circulating in Georgia, are consistent with previous studies showing the influence of strain diversity on the discriminatory power of genotyping methods [8, 10, 11]. This variation thus should be considered when choosing appropriate methodologies for molecular epidemiological studies, whereby customizing based on local strain variation is one possible way, particularly in resource-limited settings [10, 11, 21].

**Table 1:** MIRU-VNTR 24 loci set with individual HGDI and inclusion in standardized and customized genotyping panel.

Locus (24 loci panel)	12 Loci Panel	15 Loci Panel	Customized 10–loci set
MIRU 02	X		
MIRU 04	X	X	
MIRU 40	X	X	X
MIRU 10	X	X	
MIRU 16	X	X	X
MIRU 20	X		
MIRU 23	X		
MIRU 24	X		
MIRU 26	X	X	X
MIRU 27	X		
MIRU 31	X	X	X
MIRU 39	X		X
VNTR 424		X	
VNTR 577		X	
VNTR 1955		X	X
VNTR 2163b		X	X
VNTR 2165		X	
VNTR 2347			
VNTR 2401		X	X
VNTR 2461			X
VNTR 3171			
VNTR 3690		X	
VNTR 4052		X	X
VNTR 4156		X	

For increased discrimination, we additionally typed samples that were clustered based on the initial typing with the remaining loci from the 24-loci panel and re-evaluated the pre-defined customized set. The initial customized panel revealed 35.7% of clustered cases, which was subsequently reduced to 19.4% after applying an additional 14 loci. Re-evaluation of the HGDI indexes, along with assessment of the ability of each locus to identify the majority of the discrete samples during the step-by step addition to the customized 10-loci set, allowed us to arrange the remaining 14 loci into the stepwise typing approach. While the 10-loci customized set might be sufficient as a standalone panel for particular epidemiological studies, it can be also considered as a primary typing set—step 1, for the hierarchical application of additional MIRU-VNTR loci. However, if a higher discriminatory power is required, our customized stepwise approach showed the capacity to reduce the number of samples to be typed by 41.7% and 8.6% using customized/step 1 and step two typing, respectively, leading to a reduced workload, processing time and cost.

We did observe the influence of the particular study population on the discriminatory power of specific loci, for example for locus VNTR424 and VNTR3171. Therefore, evaluation of the discriminatory power by HGDI needs to consider the homogeneity of the population to be typed.

Our customized stepwise approach advances classical 24 MIRU-VNTR panel by reduced workload, processing time and direct costs (technician per-hour labor cost, consumables and reagents) by 42.6%. Given that our findings are based on local pricing of the consumables and laboratory technician average salary, the result should thus be treated with caution as they will vary based on the specific context with regards to costs of technician salaries and consumables. Variation in cost reduction (absolute number) is attributable to local pricing. In Georgia, a middle income country, the consumable prices are much higher compared to Europe and United states due to procurement and shipping complexities, whereas estimated labor costs are lower. Therefore, the direct cost savings discussed in the paper are specific for the Georgian setting but this approach is likely to result in cost savings in other settings as well.

Based on our findings, we propose “The Georgian customized 10 MIRU-VNTR loci set”, to be used for molecular epidemiological studies in the country of Georgia as a primary typing tool for identifying major clusters or defining relapse/reinfection. Moreover, the additional 14 loci could be used for further discrimination in clusters defined based on the initial typing. Such a stepwise approach using two typing sets will reduce time, cost and workload, while maintaining high discriminatory power and reproducibility in the most practical way.

Our study has several limitations. Firstly, our sample size was moderate. However, the study covered all the available samples from the penitentiary system and the combination with previous dataset from the general population had increased the variation within the sample set. Secondly, even though MIRU-VNTR typing can produce sufficient data for specific questions, if higher resolution is needed, this method can be limiting, especially in settings with a large proportion of lineage2/Beijing strains [7]. Ideally, whole genome sequencing (WGS) would be the method of choice, however it requires the high-level facility with sequencing instruments which currently are not available in Georgia. Even more importantly, the bioinformatics expertise is not widely available in Georgia, which increases complexity of using the WGS methodology. Tentatively, compared to WGS pricing in Europe and United States, the cost of the sequencing in Georgia would be much higher than MIRU-VNTR. Our study results combined with previous data showed that usage of the combination of a customized set of MIRU-VNTR loci and a stepwise approach gives useful information in the most feasible and cost-effective way.

Although conventional MIRU-VNTR is still a widely used tool, this method may still overestimate Mtb recent transmission events as confirmed by several studies [22–24]. The next decade is likely to witness transition to sequencing technologies due to considerable decrease in sequencing cost [25]. Additionally, decline of the TB burden within the country would give a significant advantage to these technologies to be used for not only epidemiological investigations, but for diagnosis and drug resistance determination as well. However, until this transition occurs in developing countries, MIRU-VNTR typing based on a customized stepwise approach could be valuable addition to the surveillance tools used in Georgia to enhance the control and prevention of TB.
